# Tapeworms in an Apex Predator: First Molecular Identification of *Taenia krabbei* and *Taenia hydatigena* in Wolves (*Canis lupus*) from Romania

**DOI:** 10.3390/pathogens15010018

**Published:** 2025-12-23

**Authors:** Maria Monica Florina Moraru, Ana-Maria Marin, Dan-Cornel Popovici, Azzurra Santoro, Adriano Casulli, Sorin Morariu, Marius Stelian Ilie, Violeta Igna, Narcisa Mederle

**Affiliations:** 1 Department of Parasitology and Parasitic Diseases, Faculty of Veterinary Medicine, University of Life Sciences “King Mihai I”, 300645 Timisoara, Romania; mariamoraru@usvt.ro (M.M.F.M.); sorinmorariu@usvt.ro (S.M.); mariusilie@usvt.ro (M.S.I.); violetaigna@usvt.ro (V.I.); narcisamederle@usvt.ro (N.M.); 2 Forestry Faculty, Transilvania University Brasov, 500123 Brasov, Romania; danpopovici30@yahoo.com; 3 WHO Collaborating Centre for the Epidemiology, Detection and Control of Cystic and Alveolar Echinococcosis (One Health), Istituto Superiore di Sanità, 00161 Rome, Italy; azzurra.santoro@iss.it (A.S.); adriano.casulli@iss.it (A.C.); 4 European Union Reference Laboratory for Parasites (EURL-P), Foodborne and Neglected Parasites Unit, Department of infectious Diseases, Istituto Superiore di Sanità, 00161 Rome, Italy

**Keywords:** *Canis lupus*, *Taenia krabbei*, *Taenia hydatigena*, Romania

## Abstract

The wolf (*Canis lupus*) is an apex predator with high mobility and trophic plasticity, serving as a valuable indicator of helminth transmission at the wildlife–livestock interface. Given the ecological overlap between wolves and both wild and domestic ungulates in Romania, we aimed to identify and molecularly characterize cestodes from wolves’ small intestines. Between November 2022 and June 2025, small intestines from nine wolves were collected across four Romanian counties, frozen, and examined using classical parasitology (macroscopic and microscopic) and molecular methods (PCR amplification and Sanger sequencing of mitochondrial *cox1*, *nad1*, and *12S rRNA* fragments). Taeniids were detected in three (33.33%) out of nine tested individuals. Genetic analyses confirmed the presence of *Taenia krabbei* and *Taenia hydatigena*—species not previously reported in wolves from Romania. This study provides the first molecular evidence of *T. krabbei* and *T. hydatigena* in wolves from Romania, and likely Eastern Europe, indicating active transmission and underscoring the need for broader surveillance of hosts to clarify their ecology and regional dynamics within a One Health context.

## 1. Introduction

Interspecific competition, defined by specialists as the predator–prey relationship, or more simply, predation, occurs between these two components on multiple levels and in a much more intimate manner, particularly in the case of parasitism, which is considered a specific form of predation [1]. Situated at the top of the trophic pyramid as opportunistic predators with high mobility, wolves serve as natural hosts for intestinal parasites (Protozoa, Trematoda, Cestoda, Nematoda), thereby facilitating their widespread dissemination across large geographical areas [2,3,4,5,6,7,8,9,10]. In the Iberian Peninsula, one of the most anthropogenically altered regions in Europe, coprological studies report a prevalence of gastrointestinal parasites (Protozoa, Trematoda, Cestoda, and Nematoda) in wolves ranging from 57.0% to 100% in Spain and from 21.5% to 68.3% in Portugal [7]. Among wolves and the ungulate species with which they interact in wild habitats, numerous ecological relationships can be identified, characterized either by the role of wolves as definitive hosts or by the role of ungulates as intermediate hosts [1].

The life cycle of taeniids, which involves two mammalian hosts—a carnivorous or omnivorous definitive host (canids, felids, mustelids, viverrids, and humans) and an intermediate host (herbivores, rodents, and lagomorphs)—represents an ideal model for studying multi-host systems and reflects epidemiological dynamics at the wildlife–livestock interface, especially where there is spatial overlap between definitive and intermediate hosts from both categories [11,12]. The life cycle of *Taenia hydatigena* entails development of the larval form, *Cysticercus tenuicollis*, on the abdominal serosae of intermediate hosts (domestic herbivores and wild ungulates), following ingestion of gravid proglottids shed in the feces of definitive hosts; in these domestic and wild carnivores, the adult tapeworm localizes in the small intestine [13,14,15,16]. *Taenia krabbei* parasitizes canids such as wolves and dogs and develops its larval form, *Cysticercus tarandi*, in the striated musculature and heart of intermediate hosts (roe deer, reindeer, and European elk) [14,17,18]. Ecological overlap between wild and domestic definitive hosts, coupled with population expansion of wildlife, raises questions regarding the spread of this cestode by one of the canid species—wolf or the dog [11]. Nevertheless, *Taenia krabbei* remains of interest to specialists primarily due not to public-health risk but to the economic impact of carcass downgrading in intermediate hosts affected by cysticerci [11].

In Romania, the wolf is the principal large carnivore species responsible for regulating proximal ungulate populations (cervids and suids) within trans-Carpathian habitats, exerting cascading ecological effects on these prey communities [19,20]. Benefiting from a robust population—one of the largest in Europe, estimated at approximately 3.000 individuals across 154.500 km^2^ [21]—Romania serves as a major stronghold for wolf conservation. This is due both to the availability of extensive wild habitats spanning all trans-Carpathian biogeographic regions, and to the species-specific conservation measures implemented at the national level [22,23,24].

Although the number of European studies dedicated to this apex predator is substantial, research conducted in the Romanian Carpathians remains limited [25]. In 2002, Gherman et al. [26] identified the following parasites in wolves: *Echinococcus granulosus*, *Toxocara canis*, *Uncinaria stenocephala*, *Ancylostoma caninum*, *Trichinella* spp., and *Linguatula serrata*.

Given the scarce reporting on the identification and distribution of cestodes in Romania’s wildlife, the aim of the present study was to provide new information on the prevalence of cestode parasitism in wolves and to molecularly characterize isolates collected from the intestines of these apex predators originating in four Romanian counties.

## 2. Materials and Methods

### 2.1. Diagnostic Procedures

**Figure 1:**
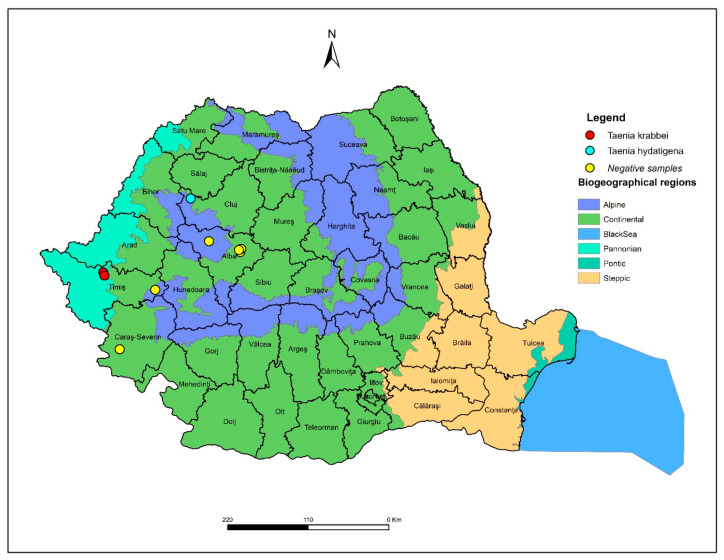
Map showing the geographical locations (counties) of the tested animals and the *T. krabbei* and *T. hydatigena* positive animals.

The research was conducted between November 2022 and June 2025 and included nine wolves (seven males and two females), aged 2–5 years. The animals originated from hunting grounds from four counties of Romania: Caraș-Severin, Timiș, Alba, and Cluj (Figure 1). For seven of the individuals, an official derogation was issued by the Ministry of Environment, Waters and Forests, in accordance with Law No. 407/2006 (on hunting and wildlife protection) [27] and Government Emergency Ordinance No. 57/2007 [28], while the remaining two individuals were found dead (roadkill). The specimens were transported to the Clinic of Parasitic Diseases within the Faculty of Veterinary Medicine, Timișoara/“King Mihai I” University of Life Sciences, Timișoara, for parasitological examination. After the examination, biosafety standards were observed in accordance with international guidelines issued by the World Organisation for Animal Health (WOAH) [29] and the European Food Safety Authority (EFSA) [30], and all nine wolf specimens were frozen at −80 °C for 48 h to inactivate agents with zoonotic potential.

#### 2.1.1. Necropsy Examination

The small intestine of each individual was placed separately in 20 L plastic containers and subsequently opened by longitudinal sectioning. Present cestodes were collected into Petri dishes containing physiological saline and examined microscopically for morphological identification. However, deep freezing had compromised helminth structural integrity, and standard morphological identification could not be performed [31]. The cestode specimens were stored in 96% ethanol for subsequent molecular identification analyses.

#### 2.1.2. Molecular Analysis

Genomic DNA was extracted from proglottids using the DNeasy Blood & Tissue Kit (Qiagen, Valencia, CA, USA), according to the manufacturer’s guidelines. A negative control containing nuclease-free water was incorporated into each DNA extraction session to monitor potential contamination. The extracted DNA was stored at −20 °C until further analysis.

A fragment of the mitochondrial Cytochrome Oxidase I (*cox1*) gene was amplified using the primers EgCOI 1 and EgCOI 2, initially described by Bowles et al. (1992) [32] and subsequently modified by Bart et al. (2006) [33]. PCR amplification was conducted in a total reaction volume of 30 μL, comprising 2 μL of DNA template, 15 μL of HotStart PCR Master Mix (Qiagen GmbH, Hilden, Germany), 0.5 μM of each primer (forward and reverse), and 10 μL of nuclease-free water. The thermal cycling profile included an initial denaturation at 95 °C for 15 min, followed by 38 amplification cycles consisting of denaturation at 94 °C for 30 s, primer annealing at 55 °C for 30 s, and extension at 72 °C for 30 s, with a final elongation step at 72 °C for 5 min.

A fragment of the mitochondrial NADH dehydrogenase subunit 1 (*nad1*) gene was amplified using the primers JB11 and JB12 [34]. PCR amplification was conducted in a total reaction volume of 30 μL, comprising 2 μL of DNA template, 15 μL of HotStart PCR Master Mix (Qiagen GmbH, Hilden, Germany), 0.5 μM of each primer (forward and reverse), and 10 μL of nuclease-free water. The thermal cycling profile included an initial denaturation at 95 °C for 15 min, followed by 35 amplification cycles consisting of denaturation at 94 °C for 30 s, primer annealing at 53 °C for one minute, and extension at 72 °C for 30 s, with a final elongation step at 72 °C for 5 min.

In addition, a fragment of the *12S rRNA* gene was amplified using primers P60 and P375, following the protocol described by Dinkel et al. (2004) [35]. PCR amplification was performed in a final reaction volume of 50 μL, comprising 5 μL of DNA template, 25 μL of HotStart PCR Master Mix (Qiagen GmbH, Hilden, Germany), 0.5 μM of each primer (forward and reverse), and 15 μL of nuclease-free water. The thermal cycling protocol consisted of an initial denaturation step at 95 °C for 15 min, followed by 40 cycles of denaturation at 93 °C for 60 s, annealing at 55 °C for 90 s, and extension at 72 °C for 2 min, with a final extension at 72 °C for 5 min. A negative control, in which nuclease-free water was used instead of a DNA template, was included in each PCR run.

PCR amplicons were visualized using capillary gel electrophoresis on the QIAxcel system (Qiagen GmbH, Hilden, Germany). Following confirmation of successful amplification, the PCR products were sent to GENEWIZ (Leipzig, Germany) for purification and subsequent Sanger sequencing. The obtained sequences were analyzed by BLAST using the NCBI online tool [36].

Sequences were deposited in GenBank under accession numbers PV857702, PV857715, and PV857721 (*cox1*), PV857764-5 (*12s*), and PV879913-5 (*nad1*).

Phylogenetic analysis was reconstructed with maximum likelihood (ML) inference using multiple *cox1* and *nad1* alignments, including the sequences obtained in this study as well as sequences available for representative Cestoda species in GenBank. The Jukes-Cantor substitution model was chosen according to the model selection function of the CLC Main Workbench 22.0.2 software (Qiagen) [37]. 1000 bootstrap replicates were performed to estimate the branch robustness.

## 3. Results

Of the nine wolf individuals examined, helminths with macroscopic characteristics consistent with the family Taeniidae were identified in three (33.33%).

PCR amplification was positive for *cox1*, *nad1*, and *12S* in two samples, and for *cox1* and *nad1* in the third sample. Sequencing of *cox1*, *nad1*, and *12S rRNA* genes confirmed the presence of *T. krabbei* in two wolves and *T. hydatigena* in one wolf. The wolves parasitized with *T. krabbei* originated from Timiș County and had intestinal burdens of 9 and 18 adult cestodes, respectively. The wolf infected with *T. hydatigena* originated from Cluj County and harbored nine tapeworms within its small intestine (Table 1).

**Figure 2:**
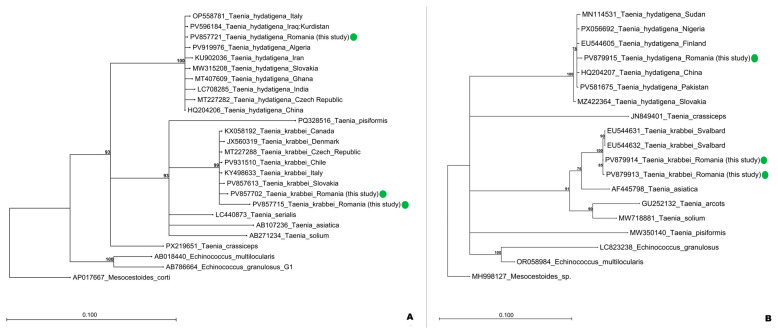
Phylogenetic relationships based on the *cox1* (380 bp) (**A**) and *nad1* (**B**) (484 bp) sequence alignments of *Taenia krabbei* and *Taenia hydatigena* obtained in this study (green dot), along with selected reference sequences from GenBank representing *T. krabbei*, *T. hydatigena*, and other related cestodes. The phylogenetic trees were constructed using the Maximum_Likelihood method in CLC Genomics Workbench, displaying only branches supported by bootstrap values >70% (1000 replicates). Reference sequences are annotated with GenBank accession numbers, scientific names, and country of origin for *Taenia krabbei* and *Taenia hydatigena*. The scale bar indicates the number of nucleotide substitutions per site.

The phylogenetic reconstruction supported the assignment of the isolates to their respective species and highlighted their evolutionary relationships with other representative taxa (Figure 2). Both *nad1* and *cox1* phylogenies placed the *T. hydatigena* isolate (PV879915 and PV857721) in a cluster with sequences from Europe, Africa and Asia, forming clades with a bootstrap value of 100, indicating robust support. This pattern suggests that the Romanian *T. hydatigena* is genetically related to European populations as well as to Asian and African samples, thus reflecting low geographic structuring within the species. The *nad1* phylogeny placed the Romanian *T. krabbei* isolates (PV879913, PV879914) in a cluster with sequences from the Svalbard archipelago (Norway), which formed a separate subcluster (90% bootstrap value). This result suggests that the Romanian sequences and those from Svalbard may belong to distinct groups, but since no additional *T. krabbei nad1* sequences are currently available in GenBank except for those from the Svalbard archipelago, it was not possible to better assess the existence of potential genetic or geographic structuring. In the *cox1* phylogeny, the Romanian *T. krabbei* isolates (PV857702, PV857715) again clustered in a single clade (99% bootstrap value); however, they show branches of different lengths, reflecting intraspecific variation also among the Romanian samples.

## 4. Discussion

This study is the first to molecularly confirm the presence of *T. hydatigena* and *T. krabbei* in wolves in Romania and, to the best of our knowledge, in Eastern Europe. Molecular analysis was made possible following the isolation of parasites from the small intestines of the examined wolf individuals. Quantification showed burdens ranging from 9 to 18 tapeworms/wolf, results comparable to those reported by Priemer (2002) [18] in Germany (1–33 tapeworms/*C. lupus*), Lavikainen (2011) [17] in Finland (1–36 tapeworms/*C. lupus*), and Al Sabi (2018) [2] in Sweden (1–11 tapeworms/*C. lupus*). *Taenia hydatigena* has been reported in wolves in Italy [14,38] and Spain [9], and it was characterized morphologically and molecularly in the wolves from Turkey [39]. *Taenia hydatigena* and *T. krabbei* have likewise been identified molecularly in wolves in Finland and Sweden [17], and in northern and western Canada [8]. Similarly, molecular identification of *T. hydatigena* and *T. krabbei* was achieved in both free-ranging wolves and those held in captivity in zoological gardens in Germany [40].

In correlation with the parasite’s biological cycle, which involves the presence of *Taenia* species in wild carnivores, either as indicators or potential sources of infection for Cervidae, a study conducted in Slovakia reports the presence of *T. hydatigena* and *T. krabbei* in wolves, in association with the identification of *Cysticercus tenuicollis* (larval form of *Taenia hydatigena*) in red deer, roe deer, and fallow deer, as well as *T. krabbei* cysticerci in the myocardium of red deer [16]. Roe deer may also act as an intermediate host for the development of *T. krabbei* muscular cysticerci, according to Formenti (2018) [11], who reported the presence of the metacestode for the first time in Italy. The molecular characterization of adult *T. hydatigena* isolated from wolves has been correlated with the first molecular confirmation of *T. hydatigena* in wild ungulates (European elk and wild boar) in Poland [41]. The first molecular identification of the *T. hydatigena* metacestode was reported in Cervidae from Peru [42], as well as in wild boar, a common prey species for wolf packs in Italy [13].

The occurrence of domestic species in wolf diets, where wild prey availability is degraded, suggests adaptive foraging shaped by resource pressure. Predator management varies among countries and is continually influenced by local, context-specific factors that modify predation rates and the intensity of human–wildlife conflict [43].

In the present study, the molecular identification of both taeniid species, *T. hydatigena* and *T. krabbei*, in wolves originating from the counties of Timiș and Cluj can be explained by the well-documented presence of wild (e.g., red deer, roe deer, fallow deer, and wild boar) and domestic (e.g., sheep, goats) intermediate hosts in these regions, which are characterized by a mosaic agro-silvatic structure and high ungulate density [44]. The territorial overlap of wolf, a species naturally present in these counties, with stable populations in the Western and Southern Carpathians, with the preferred habitats of intermediate hosts, namely the montane forests of Cluj County and the hilly areas of Timiș County, where extensive grazing and agroforestry activities are practiced, facilitates and maintains this trophic relationship between definitive and intermediate hosts [24,44,45].

By comparison, in Italy, the combination of low biodiversity of taeniid species (exclusively *T. hydatigena*, 10.5%) and the high frequency of *E. granulosus s.s.* (26.3%) may reflect a closer association between wolves and domestic animals in anthropogenically modified landscapes, compared to wild habitats [46]. In contrast, in the Czech Republic, we are witnessing a process of wolf recolonization, with the species becoming a key element in local ecological networks and in the sylvatic cycles of heteroxenous parasites, as the presence of *T. krabbei* and *T. hydatigena* has been reported in individuals from both the Central European and Carpathian populations [15]. The 33.33% prevalence of cestode infection (*T. krabbei* and *T. hydatigena*) observed in the isolates examined in the present study aligns with findings reported in Latvia, where *T. hydatigena* reaches a prevalence of 41.2% [3], as well as with data from Nearctic and Palearctic populations, where prevalence exceeds 30% [6]. Available data on cestode infection in wolves in Italy come from populations inhabiting wild habitats in northern Italy, where *T. hydatigena* reaches 19.6% and *T. krabbei* 4.5% [14], and from central Italy, where *T. hydatigena* reaches 17.6% [47]. Individuals that have crossed the Alps contributed to the recolonization of the wolf in France, where *T. hydatigena* and *T. krabbei* are present in 7.2% and 2.4% of examined wolves, respectively [10]. Coprological results from samples collected from wolves held in captivity in 14 zoological gardens across Germany highlight the role of this carnivore in the biological cycles of a broad spectrum of gastrointestinal parasites, including various Taeniidae species (3.75% prevalence) [4]. Two years later, in wolves that had recolonized German territory, the prevalence of taeniids increased to 21.74% [40]. In Poland, the presence of the genus *Echinococcus* (10%) as well as the genus *Taenia* (100%) in the wolf population represents a potential threat that should be considered in epidemiological risk assessments [48]. In contrast, the small wolf population in Sweden is unlikely to pose a significant threat to human or animal health. Results from necropsy and coprological examinations indicate reduced parasitic diversity compared to that reported in wolf populations from other regions of Europe, with *T. hydatigena* and *T. krabbei* being molecularly identified in equal proportions [2]. The identification of cestode species infecting two closely related hosts with different lifestyles (free-ranging wolves and dogs) offers new insights into the local distribution of these parasites and their potential impact on wildlife, domestic animals, and, implicitly, human health. In Switzerland, among examined wolves, the most frequently detected species was *T. hydatigena* (38.0%), whereas in domestic dogs, *T. crassiceps* predominated [49]. The wolf populations living in countries neighboring Romania (e.g., Serbia) are parasitized by a rich helminth fauna, including seven cestode species, with *T. hydatigena* reaching a prevalence of 9.8% [5].

The synthesis of these data indicates that differences in the prevalence of *T. krabbei* and *T. hydatigena* among European wolf populations primarily reflect local ecological characteristics and the ways in which these shape trophic network structures. In heavily anthropized regions, such as certain areas of Italy, the dominance of *T. hydatigena* suggests a close interaction between wolves and domestic animals, whose carcasses act as significant sources of infection. In contrast, in countries undergoing natural wolf recolonization—such as the Czech Republic—or in regions with rich wildlife communities, including Latvia, Germany, and Poland, the high prevalence and co-occurrence of both *Taenia* species indicate broad access to wild prey and the reactivation of sylvatic transmission cycles of heteroxenous parasites. Small or isolated populations, such as those in Sweden, exhibit lower parasite diversity and moderate prevalence rates, likely resulting from limited contact with a diverse range of intermediate hosts. Additionally, the transboundary mobility of wolves—for example, across the Alps into France—contributes to parasite transfer between subpopulations and to the gradual homogenization of helminth communities. Overall, the distribution patterns of the two *Taenia* species highlight a direct relationship between habitat naturalness, wild prey availability, and the degree of anthropogenic influence: *T. hydatigena* is associated with human-modified landscapes, while *T. krabbei* is characteristic of functionally intact, sylvatic ecosystems.

Tapeworms, as helminths circulating within multi-host systems, directly reflect the dietary habits of wolves, and certain species are also relevant from a *One Health* perspective [2,4,7,9,15,46].

In Romania, where predator–prey relationships frequently involve both wild ungulates and domestic animals, the presence of *Taenia* species with zoonotic potential in wolves highlights the need to strengthen parasitological surveillance and preventive veterinary measures in order to reduce the risk of transmission to economically important livestock and to human populations. These actions align with the principles of the *One Health* framework [26]. Thus, the predator–prey dynamic—specifically the relationship between wolves, wild ungulates, and domestic animals—has been documented in Romanian forests, where wild boar has been preferentially selected in the wolf’s diet, to the detriment of roe deer and red deer [24,25]. This contrasts with the situation in Poland, where predation has been reported to focus primarily on red deer and secondarily on roe deer, wild boar, and domestic animals [50,51].

Molecular characterization was essential for differentiating and confirming the species *T. krabbei* and *T. hydatigena* in wolves in Romania, and the mitochondrial *cox1*, *nad1*, and *12S* sequences obtained here extend the known eastern limit of these taeniids’ range and reveal high identity with previously reported European and North American isolates, supporting at least a pan-European distribution.

The interpretation of the results must be approached with caution, as the relatively small sample size (nine) and uneven distribution of samples (Caraș-Severin, Timiș, Alba, and Cluj counties) may influence the accurate estimation of the diversity and prevalence of the identified parasites.

## 5. Conclusions

This study provides the first molecular confirmation of *T. krabbei* and *T. hydatigena* in wolves from Romania and, to our knowledge, in Eastern Europe, indicating active transmission cycles supported by suitable intermediate hosts.

Our findings support expanded molecular surveillance of definitive (wolves, dogs) and intermediate hosts (cervids and suids) to clarify the ecology, host specificity, and regional dynamics of these cestodes within a One Health framework.

## Figures and Tables

**Table 1:** Taeniid species identified in wolves from Romania, with parasite burden, molecular markers, and GenBank accession numbers.

Wolf ID	County	Parasite Burden (*n*)	Species Identified	Molecular Markers Amplified	GenBank Accession Numbers
W1	Timiș	9	*Taenia krabbei*	*cox1, nad1, 12S*	PV857702; PV857764; PV879913
W2	Timiș	18	*Taenia krabbei*	*cox1, nad1, 12S*	PV857715; PV857765; PV879914
W3	Cluj	9	*Taenia hydatigena*	*cox1, nad1*	PV857721; PV879915

## Data Availability

The original contributions presented in this study are included in the article. Further inquiries can be directed to the corresponding author.
